# Optimal BMI cutoff points in obesity screening for Chinese college students

**DOI:** 10.3389/fpsyg.2022.1017645

**Published:** 2022-11-11

**Authors:** Zheng Wang, Jinjin Wang, Yiqin Shi, Qun Fang, Qiang Tan, Mingming Wang, Jingping Li

**Affiliations:** ^1^Soochow College, Soochow University, Suzhou, China; ^2^School of Physical Education, Soochow University, Suzhou, China; ^3^Department of Physical Education, Kangda College, Nanjing Medical University, Lianyungang, China; ^4^School of Physical Education, Qingdao University, Qingdao, China; ^5^Logistics Management Division, Soochow University, Suzhou, China

**Keywords:** college students, BMI cutoff points, ROC curve, obesity, China

## Abstract

**Objective:**

An accurate BMI classification system specific to the population is of great value in health promotion. Existing studies have shown that the BMI recommended cut-off value for adults is not suitable for college students. Thus, the current study aims to identify optimal BMI cutoff points in obesity screening for Chinese college students.

**Methods:**

Anthropometric assessments were performed on 6,798 college students (Male = 3,408, Female = 3,390) from three universities in Jiangsu, China. Exploratory factor analysis (EFA) was conducted to establish the standardized models to estimate anthropometry for male and female students. Further indices were derived from the assessments, including body mass index (BMI), relative fat mass (RFM), obesity degree percentage (OBD%), waist-to-hip ratio (WHR), waist circumference (WC), and body fat percentage (BF%). The anthropometric index with the highest correlation to the models for male and female students were selected as the gold standard for obesity screening. Receiver operating characteristic (ROC) curve was applied to evaluate diagnostic value of each anthropometric index according to the area under curve (AUC). Youden index maximum points determined the optimal cutoff points with the highest accuracy in obesity screening.

**Results:**

The anthropometric models for both male and female students consisted of three factors. Vervaeck index was selected as the gold standard for obesity screening. By comparing AUC of the anthropometric indices, we found BMI provided the highest value in obesity screening. Further analysis based on Youden index identified the optimal BMI of 23.53 kg/m^2^ for male and 23.41 kg/m^2^ for female. Compared with the universal standard recommended by World Health Organization (WHO), the adjusted BMI criteria were characterized by high sensitivity as well as specificity.

**Conclusion:**

BMI is the most appropriate anthropometric index of obesity screening for Chinese college students. The optimal cutoff points were lower than the WHO reference. Evidence substantiated the adjusted BMI criteria as an effective approach to improve accuracy of obesity screening for this population.

## Introduction

Obesity is ranked as the fifth leading cause of death globally, which has raised serious public health concerns (Safaei et al., 2021). Researchers estimated that, by 2025, global obesity prevalence would exceed 18% in men and 21% in women, of which 6% in men and 9% in women would be categorized as severe obesity (Collaboration, 2016). Obesity significantly increases risks of morbidity and mortality associated with cardiovascular disease (Khan et al., 2018), sleep disorder breathing (Peppard et al., 2000), diabetes, cancers (Bhaskaran et al., 2014; Hu et al., 2020), and musculoskeletal disorder (Jiang et al., 2011). Evidence has shown the close connection between quality of life and body weight management. Improved quality of life is evident after weight loss interventions in different age groups (Payne et al., 2018; Diao et al., 2020). Body mass index (BMI) is the most widely used measure to diagnose obesity and provide guidelines for weight loss and control in clinical practice (Seidell et al., 2001; Romero-Corral et al., 2008). World Health Organization (WHO) has defined the cutoff points of overweight and obesity for Asian populations as 23 and 25 kg/m^2^, respectively (Barba et al., 2004). To increase accuracy and generalizability of BMI classifications, an increasing number of research has examined obesity cutoff points for different populations (Rahman and Berenson, 2010; Hunma et al., 2016; Chen et al., 2018).

The collegiate period has been considered a critical time to develop lifelong healthy behaviors such as physically active lifestyle and healthy dietary patterns (Karabulut et al., 2018; Niedermeier et al., 2018; Saghafi-Asl et al., 2020). Accurate BMI classifications for college students are of great value in not only anthropometric assessment but also influence on the young generation’s behaviors in future. A recent study in Chinese college students reported that 23.5% in male and 11.9% in female were classified as either overweight or obese (Chen et al., 2020), indicating immediate attention to address the prevalent issue. The number of college students in China has been growing in the past decades and reached 44.3 million in 2022 (Ministry of Education of the People’s Republic of China, 2021). An accurate BMI standard which is specific to college students in China can make significant contributions to public health in consideration of the large population size. BMI is characterized by a number of practical advantages such as simplicity, low cost, and noninvasive measure, which make BMI an efficient screening tool for such a population size in China. However, a major concern has been noticed with respect to the precision of applying the current WHO standard to specific populations (Romero-Corral et al., 2008). Considering potential limitations of a universal BMI standard, researchers have been investigating population and ethnicity specific BMI criteria over the past decade (Hunma et al., 2016; Karabulut et al., 2018; Itani et al., 2020). The research that improves accuracy of BMI based obesity screening for Chinese college students is warranted.

The current study aims to develop an evidence-based obesity screening tool for Chinese college students. Stringent procedures were conducted to ensure robustness of the research findings. Based on a set of anthropometric measures, exploratory factor analysis (EFA) was used to establish a standardized model (z-score model) for the college students’ physique. Further analysis identified the gold standard in terms of the standardized model. The anthropometric index with the highest correlation to the z-score model was the gold standard. It is worth pointing out that the current study does not assume BMI the most appropriate measure in obesity screening. In fact, the area under the receiver operating characteristic (ROC) curve was used to identify the screening tool from available anthropometric measures. The anthropometric index with the largest area under curve (AUC) would be selected for obesity screening. Optimal cutoff points of the selected anthropometric index were calculated by the Youden index. Additionally, agreement measures of the cutoff points were assessed by Kappa index. By following the aforementioned procedures, the current study identifies the anthropometric index for obesity screening, and then determines the cutoff points of obesity.

## Materials and methods

### Participants

The current study was approved and supported by Jiangsu Physical Fitness and Health Promotion Center. The center is an official institution which administers annual fitness examination and evaluation for students in Jiangsu, China. Participants were recruited from three randomly selected universities in the province. Eligible participants must meet the following criteria: (i) undergraduate students aged between 18 and 22 years old, (ii) no chronic disease or functional impairment, and (iii) no mental health issues. Information on research purpose and procedures was acknowledged prior to the study. All participants provided written informed consent and voluntarily completed all the required tests. A total of 6,798 college students (Male = 3,408; Female = 3,390) agreed to participate in the study.

### Anthropometric measures

Height and weight were measured by a calibrated electronic scale with the precision of 0.1 cm and 0.1 kg (HM1000-SZ, HeMei Tech Corp., China). Participants were light clothing with shoes off. Participants stood in an anatomical position in the measure of standing height. Sitting height was measured as the height from the seat of the chair in which a participant was sitting to the top of the head. Participants were asked to keep the back of the head, shoulder blades and buttocks in touch with the vertical board. The thighs of the participants were touching closely together on the sitting board, forming a right angle with the trunk (Zhang and Li, 2015).

Chest, waist, and hip circumferences were measured by a nonelastic tape with a precision of 0.1 cm. For chest circumference (CC), the tape was placed at the level of the fourth rib and set snug around the body (Trüb et al., 2020). Waist circumference (WC) was measured at the midpoint between the lower edge of the rib cage and the iliac crest after a full expiration (Shrestha et al., 2021). Hip circumference (HC) was measured at the maximum protuberance of the buttocks (Skrypnik et al., 2015). Skinfold thickness was measured by a caliper in 0.1 cm. Triceps, subscapular, and abdominal site were selected for skinfold measurement. Trained research assistants measured circumferences and skinfold thickness twice for each site, with the average used for data analysis. Anthropometric measures of the participants were summarized in Table 1.

**Table 1:** Anthropometric measures of participants.

Measures	Male (*N* = 3,408)	Female (*N* = 3,390)
	*x* ± *s*	*x* ± *s*
Height (cm)	173.28 ± 5.51	160.99 ± 5.05
Weight (kg)	65.20 ± 9.90	52.74 ± 6.69
Sitting height (cm)	92.14 ± 3.46	86.42 ± 3.18
Chest circumference (cm)	86.61 ± 6.83	81.40 ± 5.73
Waist circumference (cm)	76.15 ± 8.54	69.29 ± 6.46
Hip circumference (cm)	92.68 ± 6.57	89.813 ± 5.12
Triceps skinfold (mm)	13.04 ± 6.38	17.07 ± 4.40
Subscapular skinfold (mm)	15.05 ± 6.74	16.13 ± 4.65
Abdominal skinfold (mm)	17.90 ± 9.19	19.80 ± 5.84

### Anthropometric indices

Six indices were derived from the anthropometric measures to provide further insights into the physique of college students. BMI was calculated based on body weight (BW) in kilograms (kg) and height in meters (m). The WHO recommended cutoff point for obesity is above 28 kg/m^2^ (Barba et al., 2004). BMI calculation was presented as Equation 1.


(1) $$BMI=BW/{\mathrm{Height}}^{2}$$

Relative fat mass (RFM) has been proved a valid estimator of whole-body fat percentage. Based on the measurements of height and WC in meters, the cutoff points of obesity for male and female are 22.8 and 33.9, respectively (Woolcott and Bergman, 2018). RFM for male and female was calculated as follows (Equations 2-1, 2-2):


(2-1) RFMfor male=64−20×Height/WC

(2-2) RFMfor female=76−20×Height/WC

Obesity degree (OBD) was a commonly used method for obesity screening among Chinese adults. This index is a percentage based on the calculation of BW in kg and height in cm. Individuals with OBD% above 20% are classified as obesity (Zhang et al., 2017). Calculation of OBD% was presented as Equations 3-1, 3-2.


(3-1) $$\mathrm{Standardized}\;BW=\left(\mathrm{Height}-100\right)\times 0.9$$

(3-2) $$\begin{array}{c} OBD\%=\left(BW-\mathrm{Standardized}\;BW\right)/\\ \mathrm{Standardized}\;BW\times 100\% \end{array}$$

Waist to hip ratio (WHR) was calculated by WC and HC in cm (Equation 4). Males with the WHR above 0.90 and females with the ratio above 0.85 were considered obese (Liu et al., 2018).


(4) WHR=WC/HC

Calculations of body fat percentage (BF%) consisted of a series of steps involving body density (BD) and skinfold (SF). SF was the sum of triceps and subscapular thickness in millimeters (Equation 5-1), which led to BD for male (Equation 5-2) and female (Equation 5-3). Based on BD for individuals, BF% was calculated by Equation 5-4. The cutoff points of obesity for both male and female were set at 25 and 30%, respectively (Barba et al., 2004).


(5-1) SF=Triceps thickness+Subscalpular thickness

(5-2) BDmale=1.0913−0.00116SF

(5-3) BDfemale=1.0897−0.00133SF

(5-4) $$BF\%=\left(4.570/BD-4.142\right)\times 100$$

Vervaeck index was calculated based on BW in kg, CC in cm, and height in cm (Equation 6). The index remains stable in adulthood, which makes it suitable for obesity screening among college students. The value above 94.3 was defined as obesity for Chinese (Shang et al., 2007).


(6) $$\mathrm{Vervaeck index}=\left(BW+CC\right)/\mathrm{Height}\times 100$$

### Statistical analysis

EFA was used to extract key factors of the standardized anthropometric model (z-score model) for college students. Varimax orthogonal rotation was conducted to examine model fit and calculate factor loadings. The factors were identified based on eigenvalues, factor loadings, and the interpretability of the extracted factors. The gold standard for obesity screening was selected from the anthropometric indices with the highest correlation to the standardized model. The ROC curve was drawn by MedCalc 18.2. Area under curve (AUC) reflects the diagnostic value, with 0.5<AUC ≤ 0.7 for low diagnostic value, 0.7<AUC ≤ 0.9 for medium diagnostic value, and AUC>0.9 for high diagnostic value (Dou et al., 2016). The anthropometric index with the highest diagnostic value would be chosen as the tool for obesity screening. Youden index maximum points helped to identify the optimal cutoff points for obesity screening. Kappa index was used as agreement measures to testify and improve accuracy of the cutoff points in screening obesity. The significance level was set at the value of *p* of 0.05. All statistical analyses were conducted by SPSS 25.

## Results

EFA identified three primary factors of the anthropometric model for both male and female college students. The three factors accounted for 83.61 and 78.09% of the variance in the anthropometric data for male and female, respectively. Factor 1 (z_1_) consisted of BW, CC, WC, and HC. Factor 2 (z_2_) identified three skinfold measures of triceps, subscapular, and abdominal thickness. Factor 3 (z_3_) included two measures of height which were standing height and sitting height. Factor loadings of the extracted anthropometric measures were above 0.7. The standardized (z-score) models for male and female students were presented as Equations 7-1, 7-2, respectively. Table 2 indicated factor loadings of the anthropometric measures for male and female students.

**Table 2:** Rotated factor loading of the anthropometric measures.

Measures	Male			Female		
	Factor 1	Factor 2	Factor 3	Factor 1	Factor 2	Factor 3
Height (cm)	0.202	−0.050	**0.866**	0.223	−0.121	**0.867**
Weight (kg)	**0.830**	0.301	0.299	**0.786**	0.272	0.347
Sitting height (cm)	0.056	0.018	**0.905**	0.049	0.102	**0.903**
Chest circumference (cm)	**0.865**	0.292	0.085	**0.864**	0.237	0.073
Waist circumference (cm)	**0.823**	0.449	0.014	**0.869**	0.192	−0.034
Hip circumference (cm)	**0.805**	0.328	0.151	**0.816**	0.213	0.234
Triceps skinfold (mm)	0.315	**0.854**	−0.025	0.193	**0.817**	0.013
Subscapular skinfold (mm)	0.345	**0.860**	−0.006	0.435	**0.733**	−0.051
Abdominal skinfold (mm)	0.354	**0.854**	0.001	0.159	**0.863**	0.035

The bold numbers highlight the anthropometric measures in accordance with one of the factors of the standardized model for male and female students.


(7-1) $$Z_{\mathrm{male}}=41.857\%z_{1}+35.682\%z_{2}+22.461\%z_{3}$$

(7-2) $$Z_{\mathrm{female}}=43.958\%z_{1}+31.096\%z_{2}+24.947\%z_{3}$$

Vervaeck index was chosen as the gold standard because of the highest correlation to the z-score model. The coefficients for male and female were *r* = 0.847 and *r* = 0.817, respectively. Table 3 summarized the correlation coefficients of anthropometric indices to the z-score models.

**Table 3:** Correlation coefficients of anthropometric indices to the *z*-score model.

Indices	Correlation to *z*-score model	
	Male	Female
Vervaeck index	0.847^**^	0.817^**^
OBD%	0.761^**^	0.643^**^
RFM	0.771^**^	0.602^**^
WHR	0.544^**^	0.333^**^
WC	0.887^**^	0.767^**^
BMI	0.798^**^	0.740^**^
BF%	0.815^**^	0.713^**^

**p < 0.001; OBD%, obesity degree percentage; RFM, relative fat mass; WHR, waist-to-hip ratio; WC, waist circumference; BMI, body mass index; BF%, body fat percentage.

**Figure 1:**
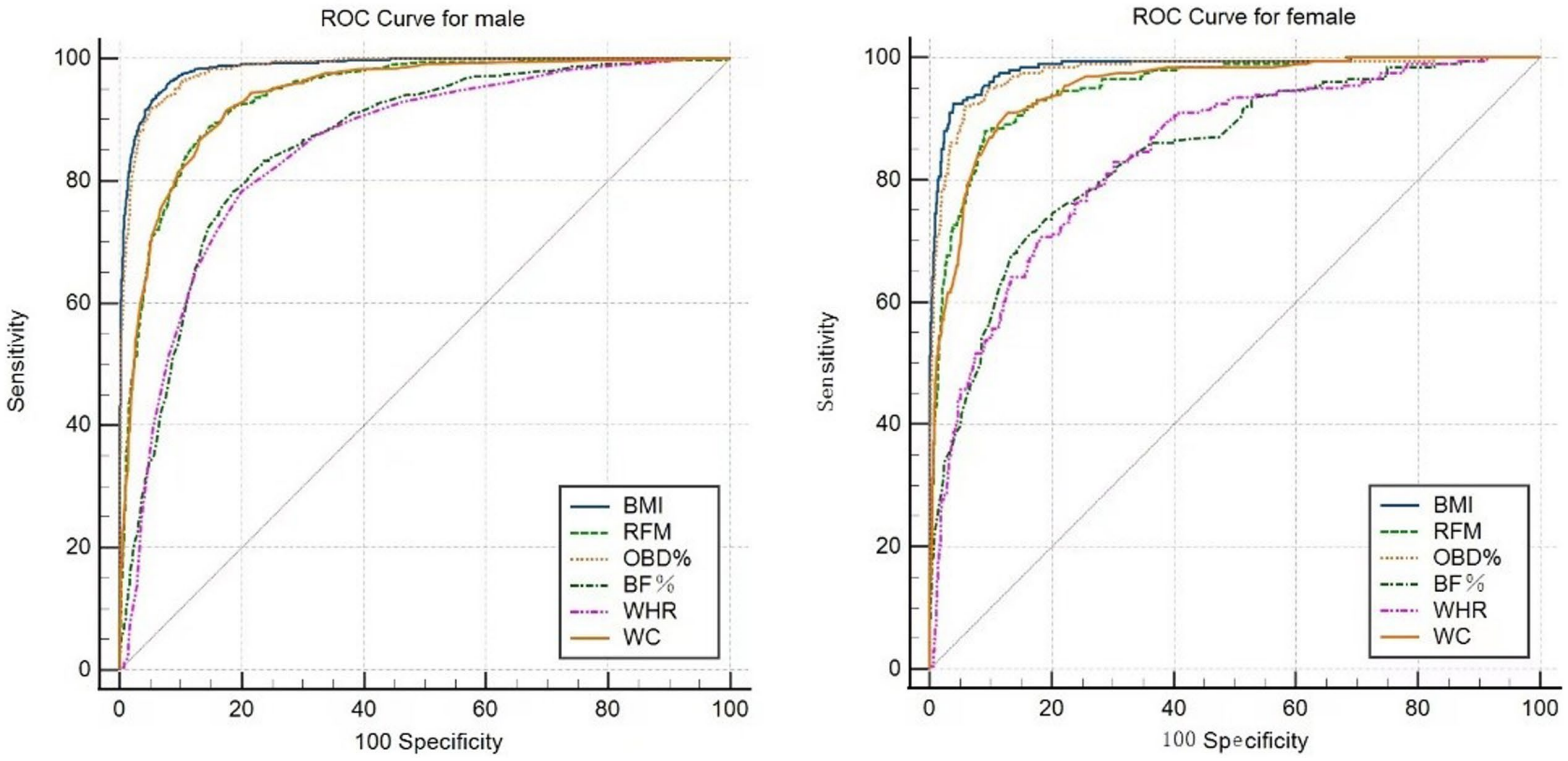
ROC curves for male (left) and female (right).

ROC curves display the accuracy of BMI, OBD%, RFM, WC, BF%, and WHR in obesity screening for male and female students (Figure 1). With Vervaeck index as the gold standard, the mean AUC of BMI was greater than that of other factors for both male (AUC = 0.986, 95% CI: 0.981–0.990) and female students (AUC = 0.983, 95% CI: 0.979–0.988), suggesting the best accuracy of using BMI to diagnose obesity for the college students. The mean AUC of each anthropometric index was listed in Table 4.

**Table 4:** AUC by the anthropometric indices.

Indices	Male	Female
BMI	0.986 (0.981–0.990)	0.983 (0.979–0.988)
RFM	0.939 (0.931–0.947)	0.949 (0.941–0.956)
OBD%	0.983 (0.978–0.987)	0.977 (0.971–0.981)
WHR	0.853 (0.840–0.864)	0.838 (0.825–0.850)
WC	0.939 (0.931–0.947)	0.949 (0.941–0.956)
BF%	0.859 (0.847–0.871)	0.844 (0.832–0.856)

OBD%, obesity degree percentage; RFM, relative fat mass; WHR, waist-to-hip ratio; WC, waist circumference; BMI, body mass index; BF%, body fat percentage.

The optimal cutoff points of BMI for obesity screening were determined by the maximum point of Youden index. The BMI cutoff point for male students is 23.53 kg/m^2^ in corresponding to the Youden index of 0.877. Compared with the WHO reference of 28 kg/m^2^, the optimal cutoff point shows prominent improvement in sensitivity from 19.91 to 93.77% and decline in specificity from 99.96 to 93.93% (Table 5). The Youden index for female students is 0.8726 in corresponding to the optimal BMI cutoff point of 23.41 kg/m^2^. Compared with the WHO reference, the adjusted value shows an increased sensitivity from 14.63 to 91.22% and a drop in specificity from 100 to 96.04%. The optimal BMI cutoff points indicate a remarkable improvement in the sensitivity associated with a high level of specificity for both male and female college students (Table 5).

**Table 5:** Sensitivity and specificity of original and adjusted BMI cutoff points.

Sex	Original cutoff points (kg/m^2^)			Adjusted cutoff points (kg/m^2^)			
	Reference	Sensitivity (%)	Specificity (%)	Youden index	Adjustment	Sensitivity (%)	Specificity (%)
Male	≥28	19.91	99.96	0.877	≥23.53	93.77	93.93
Female	≥28	14.63	100.00	0.873	≥23.41	91.22	96.04

Adjusted BMI cutoff points largely increased agreement to the gold standard (Vervaeck index) for obesity screening, which was evident by significant improvement in Kappa index for male (Original cutoff point = 0.289, adjusted cutoff point = 0.817) and female students (Original cutoff point = 0.251, adjusted cutoff point = 0.697). Statistics of Kappa index associated with the adjusted BMI cutoff points were listed in Table 6.

**Table 6:** Kappa statistics of original and adjusted BMI cutoff points.

		Kappa	SD	*t*	Value of *p*
Original BMI	Male	0.289	0.020	23.921	<0.001
	Female	0.251	0.037	22.047	<0.001
Adjusted BMI	Male	0.817	0.012	48.014	<0.001
	Female	0.697	0.024	41.684	<0.001

The adjusted BMI cutoff points classified higher proportion of college students into obesity than the WHO reference. According to the new BMI cutoff points, 23.00% of male and 9.29% of female students were identified as obesity, while the original criteria (BMI ≥ 28 kg/m^2^) only accounted for 3.93 and 0.91% of male and female students, respectively (Table 7). The results suggest that the adjusted BMI be more powerful in obesity screening and effectively lower the risk of false classifications.

**Table 7:** Comparisons between original and adjusted BMI cutoffs in obesity screening.

		Obesity	Non-obesity	Obesity %
Original BMI	Male	134	3,274	3.93
	Female	31	3,359	0.91
Adjusted BMI	Male	784	2,624	23.00
	Female	315	3,075	9.29

## Discussion

The current study explored the optimal BMI cutoff points for obesity screening among Chinese college students. Compared with the WHO standard for general population (BMI ≥ 28 kg/m^2^), the cutoff points for Chinese male (BMI ≥ 23.53 kg/m^2^) and female college students (BMI ≥ 23.41 kg/m^2^) were lower. The reduced BMI cutoff points were characterized by high sensitivity and specificity as well as good consistency with the gold standard based on Vervaeck index, which substantiated the new criteria in obesity screening. According to the adjusted BMI standard, an increased number of students were classified as obesity.

The evidence indicated that the original cutoff point may be too high to accurately reflect obesity prevalence among college students in China. Indeed, prominent differences across populations implied the necessity of developing population-specific criteria for obesity screening (Deurenberg, 2001). The use of BMI cutoff points for classifying obesity should account for ethnicity given that a universal BMI standard may be not appropriate in clinical practice (Norgan, 1994). Cumulative evidence suggests that, compared with the WHO reference, lower BMI values should be applied particularly in Asians (Mascie-Taylor and Goto, 2007). Nguyen and colleagues provided evidence for lower BMI cutoff point in Chinese adults than that in Western populations (Nguyen et al., 2008). Such a conclusion was substantiated by the research in which Taiwan Chinese were characterized by lower BMI associated with higher BF% than Caucasians (Chang et al., 2003). Further analysis indicated that the BMI obesity cutoff point of 30 kg/m^2^ for Caucasians was comparable to 25 kg/m^2^ for Taiwan Chinese. Consistent evidence can be found in a study involving Hong Kong Chinese (Ko et al., 2001). Researchers identified a BMI of 26 kg/m^2^ in corresponding to obesity defined by BF%.

Education is another important consideration in obesity classification. Research has shown that obesity is more prevalent among the low educated individuals compared with college graduates (Cohen et al., 2013). The lower BMI cutoff points identified in the current study can be justified by the inverse relationship between education level and obesity (Hermann et al., 2011; Boing and Subramanian, 2015). People with higher educational attainment may be better aware of the consequences of obesity and approaches to a healthy lifestyle through restricted diet and regular exercise (Eide and Showalter, 2011). In addition, social network also has substantial impact on health-related behaviors (Kim, 2016). The networks formed by individuals with higher educational attainment may provide financial, physical, and emotional support for health promotion (Berkman, 1995). It is reasonable to apply lower BMI classifications to obesity screening among college students compared with general populations.

In developed countries the rate of obesity in females is 1.5 to 2 times as many as that in males (Seidell, 2005). The current study also identified difference between male and female students, but the rate of obesity in male students (23.00%) is over twice as many as that in females students (9.29%). It is worth noting that preferences for physical appearance impose particular influences on females in Asian countries (Jackson et al., 2016). In China, more female college students are categorized as normal weight and underweight than male counterparts (Chen et al., 2020). An epidemic study in obesity prevalence in urban adults of Northeast China also identified higher obesity rate in males than that in females (Wang et al., 2012). It is worth pointing out potential sociocultural influence on body image among Chinese, as females reported greater body dissatisfaction than males. While males consider increasing muscle mass and weight essential to enhance body image, females show particular interests in body weight management (Xu et al., 2010). A recent study identified a quadratic relationship between female BMI and attractiveness ratings. Young females perceived a BMI of 22.00 ideal for body attractiveness, which was lower than the BMI preference (BMI = 25.75) in males (Han et al., 2021). The distinct perceptions on body image led to different body change behaviors between males and females in China, which could interpret the lower BMI and obesity prevalence among females.

The current study identified high sensitivity (93.77% for male and 91.22% for female) as well as specificity (93.93% for male and 96.04% for female) of the optimal BMI cutoff points in obesity screening for college students. However, the poor sensitivity has been considered a limitation of the BMI classifications in previous research. Sensitivity of the WHO reference (BMI ≥ 30 kg/m^2^) for older adults was only 14.5% for male and 23.4% for female, indicating poor efficacy of identifying obesity in this population (Batsis et al., 2016). In another research on Chinese children and adolescents, the sensitivity of BMI references for obesity varied between 12.8 and 47.3%, indicating limited accuracy of diagnosis (Chen et al., 2018). A meta-analysis provided robust evidence for accuracy of commonly used BMI values for obesity screening. The results reported a sensitivity of 50%, indicating that half of individuals with excess BF% failed to be identified based on the BMI classifications (Okorodudu et al., 2010). Therefore, increasing the sensitivity of BMI is needed to reduce the false negative rate in obesity screening (Fu et al., 2003).

The lower BMI cutoff points of the current study resulted in a higher sensitivity, which was substantiated by the previous research involving Saudi adult population. Compared with the BF%-defined obesity (83.9% in men and 97.3% in women), the BMI cutoff point of 30 kg/m^2^ classified only 29% of men and 53% of women as obesity. The BMI sensitivity reached a comparable level to the BF% classification as the BMI cutoff points were reduced to 24 kg/m^2^ (Alammar et al., 2020). It is reasonable to adopt a more stringent standard with lower BMI cutoff points for the concerns with the low BMI sensitivity in obesity screening (Javed et al., 2015). The current study proposed the BMI references with high sensitivity as well as specificity which may be attributed to age of the participants due to the strong correlation between age and body fatness (Gallagher et al., 1996). Other factors such as the gold standard for reference, prevalence of obesity, and populations can also impact specificity and sensitivity of BMI (Leeflang et al., 2013; Gába and Přidalová, 2016).

The current study has some specific contributions to the field of public health. Existing research on Chinese college students is limited, which demands practical and reliable approach to identify obesity prevalence for this population. Exercise intervention during an individual’s college period is particularly important for obesity prevention in lifetime. The lower BMI cutoff points with high sensitivity for obesity screening would facilitate obesity prevention for college students in China. The new BMI cutoff points increased the number of college students who were not considered obese by the commonly used BMI reference. It is possible that a few students may be falsely categorized as obesity, but the benefits associated with the higher sensitivity would exceed potential costs in corresponding to the increased obesity prevalence. Compared with previous research which selected bioelectrical impedance analysis of body composition and dual-energy x-ray absorptiometry as the gold standard (Anderson et al., 2012), the current study chose Vervaeck index which was convenient to use in clinical practice. BMI-based assessment allows quick and non-invasive applications to a large population. Precise cutoff points specific to Chinese college students are crucial for obesity screening and prevention.

## Conclusion

Compared with other anthropometric measures, BMI is the best approach of obesity screening for Chinese college students. The optimal cutoff points for both male and female students are lower than the WHO reference, leading to a higher proportion of obesity. Prominent increase in sensitivity was identified along with high level of specificity in the adjusted BMI, which substantiated applications of the new cutoff points to obesity screening. The current study provides health policy implications. On the one hand, the population-based cutoffs improve screening accuracy in clinical practice. In addition, the findings highlight the feasibility of implementing a stricter BMI standard for college students in China.

## Data availability statement

The raw data supporting the conclusions of this article will be made available by the authors, without undue reservation.

## Ethics statement

Ethical review and approval was not required for the study involving human participants in accordance with the local legislation and institutional requirements. Written informed consent to participate in this study was obtained from the participants.

## Author contributions

ZW and QF: Conceptualization, methodology, and writing - original draft preparation. ZW and JL: Validation. JL, QT, and JW: Investigation. ZW, YS, JL, and JW: Resources. ZW and MW: Data curation. QF: Writing - review and editing. All authors have read and agreed to the published version of the manuscript.

## Funding

This research was funded by the Jiangsu Research Center for Student Physical Health Promotion, grant number 2019KT009.

## Conflict of interest

The authors declare that the research was conducted in the absence of any commercial or financial relationships that could be construed as a potential conflict of interest.

## Publisher’s note

All claims expressed in this article are solely those of the authors and do not necessarily represent those of their affiliated organizations, or those of the publisher, the editors and the reviewers. Any product that may be evaluated in this article, or claim that may be made by its manufacturer, is not guaranteed or endorsed by the publisher.
